# Impact of high dietary energy on obesity and oxidative stress in domestic pigeons

**DOI:** 10.1002/vms3.478

**Published:** 2021-04-03

**Authors:** Seyedeh Alemeh Hosseinian, Fereshteh Hasanzadeh

**Affiliations:** ^1^ Department of Clinical Science School of Veterinary Medicine Shiraz University Shiraz Iran

**Keywords:** dietary energy, obesity, oxidative stress, pigeon

## Abstract

Obesity is associated with increased risk of oxidative stress in humans and laboratory animals but information regarding obesity‐induced oxidative stress in birds is lacking. Therefore, this study aimed to investigate the influence of high‐energy diets (HED) on obesity and oxidative stress in domestic pigeons. Forty‐five adult clinically healthy‐domestic male pigeons were randomly assigned to three equal dietary groups including low (2,850 kcal/kg), medium (3,150 kcal/kg) and high (3,450 kcal/kg) energy diets (named low energy diet, medium‐energy diet and HED, respectively). All birds received formulated diets for 60 consecutive days. Several parameters such as feed intake, body weight (BW), average weight gain (AWG) and total weight gain were determined. Serum concentrations of triglyceride (TG), total cholesterol (TC), high‐, low‐ and very‐low‐density lipoprotein cholesterols, alanine aminotransferase (ALT), aspartate aminotransferase (AST) and alkaline phosphatase (ALP) were evaluated at days 0, 30 and 60; and serum levels of total antioxidant capacity (T‐AOC), malondialdehyde (MDA) and cortisol were also measured at day 60. On day 60, five pigeons from each group were randomly euthanized and some parameters such as weight and relative weight of liver, breast muscle, and abdominal fat were determined. Furthermore, hepatic total fat content was also evaluated. BW, AWG, total weight, and circulating TG, TC, ALT, AST, ALP, MDA and cortisol in HED were significantly higher than other groups. Serum T‐AOC in HED was significantly lower than the other groups. In conclusion, this study showed that increasing dietary energy up to 3,450 kcal/kg in pigeons led to obesity and oxidative stress in them. Accordingly, it could be stated that HED and obesity induce oxidative stress in pigeon and controlling the dietary energy intake of pigeons is necessary to prevent oxidative stress in them.

## Abstract 2

The results of the current research suggested that obesity is associated with oxidative stress in pigeons.

## 1 INTRODUCTION

Over the last several years, the prevalence of obesity is increasing in avian species (Cherian, 2007; Coogan et al., 2018). Several dietary and non‐dietary reasons, such as consuming of caloric‐dense diets, high‐fat seeds and lack of physical activity contribute extensively for the inception of obesity and obesity‐associated metabolic syndrome in avian, especially companion birds (Shini et al., 2020). Sunflower and flaxseed, containing at least 40%–50% fat, are a major part of the diet in some birds like parrots and canaries (Goyal et al., 2014). Chronic consumption of high‐fat seeds can be led to excess energy intake, obesity and dyslipidaemia in birds (Najafi et al., 2020). Obesity is associated with an increased risk of fatty liver, cardiovascular diseases and sudden death in birds (Cherian, 2015). The study of obesity is valuable for further understanding its pathophysiology and management. In recent years, various studies have been designed on experimental obesity in birds (Dong et al., 2015).

To date, high‐fat diets (HFDs) have been commonly used to induce obesity in avian species (Recena Aydos et al., 2019). It has been shown that HFD in broilers and layer hens is associated with weight gain, dyslipidaemia and high‐fat accumulation in body tissues (Nikravesh‐Masouleh et al., 2018). Meleg et al., (2000) reported that 2,850 kcal/kg is a standard and suitable dietary energy for optimal growth and health status in pigeons. Bu et al. (2015) used 3,150 kcal/kg to induce fatty liver in adult pigeons. They showed that increasing dietary energy up to 3,150 kcal/kg led to increased liver weight, but did not led to develop fatty liver in pigeons. In recent years, various studies have been performed on the association between obesity and oxidative stress in humans and laboratory animals (Duan et al., 2018; Gómez‐Elías et al., 2019). It has been established that obesity and dyslipidaemia led to oxidative stress in human beings (Sumida et al., 2013). Poljsak et al., (2013) stated that HFD led to oxidative stress in rat through high reactive oxygen species (ROS) generation, lipid peroxidation and decrease antioxidant enzyme production (Poljsak et al., 2013).

During oxidative stress, an imbalance between ROS production and antioxidant defense mechanisms occurs, which is shifted towards overgeneration of ROS and oxidizing status (Rahal et al., 2014). ROS affect harmfully on various cellular structures like lipid, protein and nucleic acid that can lead to cellular dysfunction and the development of metabolic disorders (Rahal et al., 2014). This situation can be assessed by evaluating serum concentrations of oxidative stress biomarkers and the measuring of total antioxidant capacity (T‐AOC) is a common route for assessment of oxidative status (Rahal et al., 2014). Although the link between obesity and oxidative stress in humans and animals has been proven. However, there is low available information about the effects of HFD on oxidative stress in birds. Therefore, this study aimed to investigate the influence of three different levels of dietary energy consumed for 60 consecutive days on obesity and its related parameters in domestic pigeons. The main purpose of this research was the evaluation of the effects of obesity on oxidative stress in this species. The results of this research may aid to better understand the pathophysiologic effects of obesity on health status of birds.

## 2 MATERIALS AND METHODS

### 2.1 Birds and experimental design

Forty‐five adult clinically healthy‐domestic male pigeons (*Columba livia domestica*) weighting 345 ± 15 g were entered to this study in February 2019. The experiment lasted for eight weeks and throughout the experiment, all groups were housed in air‐conditioned room (24 ± 2°C) with 55% humidity on a 12 hr light/dark cycle and water and feed were ad libitum. General health and mortality were recorded continuously throughout the experiment. All pigeons were kept under similar conditions and fed a basal diet for 15 days, as an adaptation period. Then, they were randomly assigned to three equal dietary groups including control or low energy diet (LED), medium‐energy diet (MED) and high‐energy diet (HED). LED, MED and HED fed with a basal diet containing 2,850 (Meleg et al., 2000), 3,150 and 3,450 kcal/kg metabolizable energy (ME) for eight weeks, respectively. Diets were formulated based on corn and soybean meal to meet the nutrient recommendations for pigeons (Sales & Janssens, 2003) as described in Table 1.

**TABLE 1:** Ingredients and chemical composition of different used diets in the study (as fed basis)

Items	Groups		
	LED	MED	HED
Chemical composition			
Metabolizable energy (kcal/kg)	2,850	3,150	3,450
Crude protein (%)	15.04	15.05	15.06
Crude fat (%)	2.2	4.5	10
Ingredients (g/100 g, as fed)			
Corn	53.44	56.01	57.32
Soybean meal	14.41	13.85	13.93
Fat	1.53	4.8	8.28
Gluten meal	4.80	4.65	4.51
Wheat bran	12.15	10.50	8.23
Ca%22P%18	1.72	1.78	1.59
Oyster	1.30	1.25	1.28
Zeolite	6.85	3.45	1.21
Calcium carbonate	1.3	1.3	1.3
NaCl	0.30	0.31	0.31
Lysine	0.75	0.75	0.70
Threonine	0.8	0.8	0.8
Methionine	0.45	0.35	0.34
Premix^a^	0.2	0.2	0.2

Abbreviations: HED, high‐energy diet; LED, low‐energy diet or control group; MED, medium‐energy diet.

^a^ Vitamin and mineral content per kilogram of premix: vitamin A: 3,600,000 IU; vitamin D3: 800,000 IU; vitamin E: 7,200 IU; vitamin K3: 0.8 g; vitamin B1: 0.71 g; vitamin B2: 2.64 g; vitamin B3: 3.92 g, vitamin B5: 11.88 g; vitamin B6: 1.176 g; vitamin B12: 6 mg; folic acid: 0.4 g; biotin: 40 mg; choline chloride: 100 g; selenium: 80 mg; cobalt: 100 mg; iodine: 396 mg; copper:4 g; zinc: 33.88 g; iron: 20 g; manganese: 39.68 g.

### 2.2 Feed intake, body weight and weight gain

Daily food consumption was recorded to determine feed intake (FI) in each group. FI per group was measured over a 2‐week intervals (0–2, 2–4, 4–6 and 6–8 weeks). The feed was usually weighed at feeding time and then the remaining food in the feeder was weighed at the end of second week. FI was calculated by subtracting feed residues in the feeder from feed offered to each group in mentioned four period times. At the end of experiment (day 60) total FI was calculated in each group. All pigeons were weighed before starting the experiment (at day 0). Then, during the experiment, all birds were individually weighed on weeks 2, 4, 6 and 8 to determine average weight gain (AWG). The AWG per bird was calculated over a 2‐week interval (0–2, 2–4, 4–6 and 6–8) by subtracting the body weight (BW) at the end of the second week from the initial BW. At the end of the experiment, the final BW (on day 60) was subtracted from the initial BW (on day 0) to calculate the total weight gain (TWG) per bird in each group.

### 2.3 Blood sampling and biochemical assays

Blood samples (3 ml) were collected from the wing vein of 10 pigeons in each group, on days 0, 30 and 60, after 10 hr fasting. Blood samples were collected using 23‐gauge needles into the plain glass tubes, centrifuged at 750× g for 15 min at room temperature to harvest the serum. Then, the collected sera were stored at −22°C until future biochemical analysis. The serum levels of triglyceride (TG), total cholesterol (TC), high‐, low‐ and very‐low‐density lipoprotein cholesterols (HDL‐C, LDL‐C and VLDL‐C) were measured by an automatic biochemical analyzer (Biotecnica, Targa3000) using commercial investigation kits (Pars Azmoon^®^ Kit). The sera were analysed for activity of alanine aminotransferase (ALT), aspartate aminotransferase (AST) and alkaline phosphatase (ALP) using commercial kits (Pars Azmoon^®^ Kit) by an automatic biochemical analyser. At the end of experiment (day 60), the level of T‐AOC and malondialdehyde (MDA) as oxidative stress biomarkers were measured in collected sera. T‐AOC assayed by spectrophotometric method (Kiazist Life Sciences CO; sensitivity equal to 20 nmol/ml; intra‐assay and inter‐assay CV < 17% and 16%, respectively). MDA was analysed by colorimetric/fluorometric method (Kiazist life science CO; sensitivity equal to 10 mM; intra‐assay and inter‐assay CV < 15% and 19%, respectively). On day 60, the serum concentration of cortisol was evaluated by ELISA technique using the commercial kits (DIMETRA) according to the instructions of the manufacturer of the kit.

### 2.4 Bird sacrifice and sample collection

On day 60, five pigeons from each group were randomly selected and slaughtered followed by weighting and bleeding. The birds were euthanized by decapitation, different organs including the liver, breast muscle and abdominal fat were removed and weighted individually from each selected bird. Relative organ weights (% BW) was calculated by the following formula (Peter et al., 2018): Relative organ weights ꞊ [organ weight (g)/BW (g)] × 100.

In addition, from each sacrificed pigeons, one appropriate sample from the liver was harvested and immediately transported to a −80°C freezer until future laboratory analysis. The total fat content of harvested liver samples was extracted and purified based on the method of Hara and Radin (1978) with some modification suggested by Aminlari et al. (2019). Briefly, 18 ml of hexane:isopropanol (3:2) was added to 1 g of liver sample; the mixture was homogenized (DI 18B; IKA) at 5,000 rpm for 30 s and maintained overnight at 4°C. The mixture was centrifuged at 5,000× g for 20 min. Non‐lipids in the extract were removed by mixing the supernatant two times with 12 ml of aqueous sodium sulphate (prepared from 1 g of the anhydrous salt and 15 ml of water). Two layers formed in tubes and the lipids were in the upper, hexane‐rich layer. Then, the TG and TC levels of the lipid extract were analysed using commercial kits according to the manufacturer's instructions (Pars Azmoon^®^ Kit).

### 2.5 Statistical analysis

The data were analysed employing one‐way ANOVA (SPSS version 22.0) and subjecting them to the post hoc Tukey honestly significant difference (HSD) test. In cases of a failed normality test, Kruskal–Wallis ANOVA was performed followed by Dunn's post hoc test. All values were expressed as the mean and standard error (mean ± *SE*), and the value of *p* <.05 was used as statistical significance. All data presented are three replicates. All figures were drawn using GraphPad Prism 6.0 (Graph Pad Inc.).

## 3 RESULTS

The effect of various dietary energy levels on BW, FI, AWG and TWG of pigeons is presented in Table 2. The HED group showed significantly a higher BW than other groups during the trial (*p* <.05). Increasing the dietary energy content in HED group positively affected the final BW and TWG (*p* <.05). As Table 2 shows, HED and MED had significantly lower FI (g) throughout the experiment compared to the control group (LED).

**TABLE 2:** Bodyweight, average and total weekly weight gain, and average daily feed intake (gram; mean ± *SE*) of pigeons fed by three levels of dietary energy

Parameters	Groups		
	LED	MED	HED
Body weight			
0 w	353.25±16.35^a^	329.02±18.92^b^	365.87±15.08^a^
2 w	355.62±12.60^b^	340.62±12.60^b^	373.10±10.22^a^
4 w	356.37±11.85^b^	351.25±12.52^b^	399.75±7.41^a^
6 w	375.62±9.32^b^	381.25±15.86^b^	423.37±6.77^a^
8 w	377.35±14.46^b^	404.37±16.42^b^	445.25±13.64^a^
Weekly weight gain/bird			
0‐2 w	0.92±0.01^b^	9.19±1.02^a^	7.82±1.03^a^
2‐4 w	0.74±0.03^c^	10.65±2.03^b^	25.95±3.79^a^
4‐6 w	19.25±2.06^b^	30.04±5.28^b^	23.82±2.91^a^
6‐8 w	1.68±0.02^c^	22.95±6.14^b^	21.40±10.09^a^
Daily feed intake/group			
0‐2 w	336.01	218.50	168.01
2‐4 w	480.21	210.63	190.69
4‐6 w	440.24	242.62	238.56
6‐8 w	535.31	229.23	210.59
Total weight gain/bird	21.48±3.87^c^	65.37±8.02^b^	72.06±5.25^a^

^a,b,c^Different letters in the superscripts of the same row indicate significant differences (*p* < .05).

Abbreviations: HED, high energy diet; LED, low energy diet or control group; MED, medium energy diet.

Table 3 represents the weight and relative weight of some organs (liver, abdominal fat and breast muscle) in the various treatment groups. The average weight (g) and the relative weight (%) of the liver and abdominal fat mass were significantly higher in the HED group than in LED and MED (*p* <.05). The average weight (g) of the breast meat was higher in HED than in LED (*p* <.05).

**TABLE 3:** The weight and relative weight of abdominal fat, liver and breast muscle (mean ± *SE*) of pigeons fed by three different dietary energy levels on days 60

Organs	Groups		
	LED	MED	HED
Weight (g)			
Abdominal fat	3.70 ± 0.20^a^	5.40 ± 0.08^b^	6.86 ± 0.52^c^
Liver	6.68 ± 0.22^a^	6.88 ± 0.61^a^	8.55 ± 0.92^b^
Breast muscle	15.08 ± 0.10^a^	18.53 ± 0.18^b^	23.71 ± 0.15^c^
Relative weight (%)			
Abdominal fat ratio	0.98 ± 0.01^a^	1.30 ± 0.01^b^	1.54 ± 0.01^c^
Liver ratio	1.77 ± 0.01^a^	1.66 ± 0.00^a^	1.92 ± 0.01^b^
Breast muscle ratio	3.99 ± 0.07^a^	4.47 ± 0.09^b^	5.32 ± 0.04^c^

Note

Different letters in the superscripts (^a,b,c^) of the same row indicate significant differences (*p* <.05).

Abbreviations: HED, high‐energy diet; LED, low‐energy diet or control group; MED, medium‐energy diet.

**FIGURE 1:**
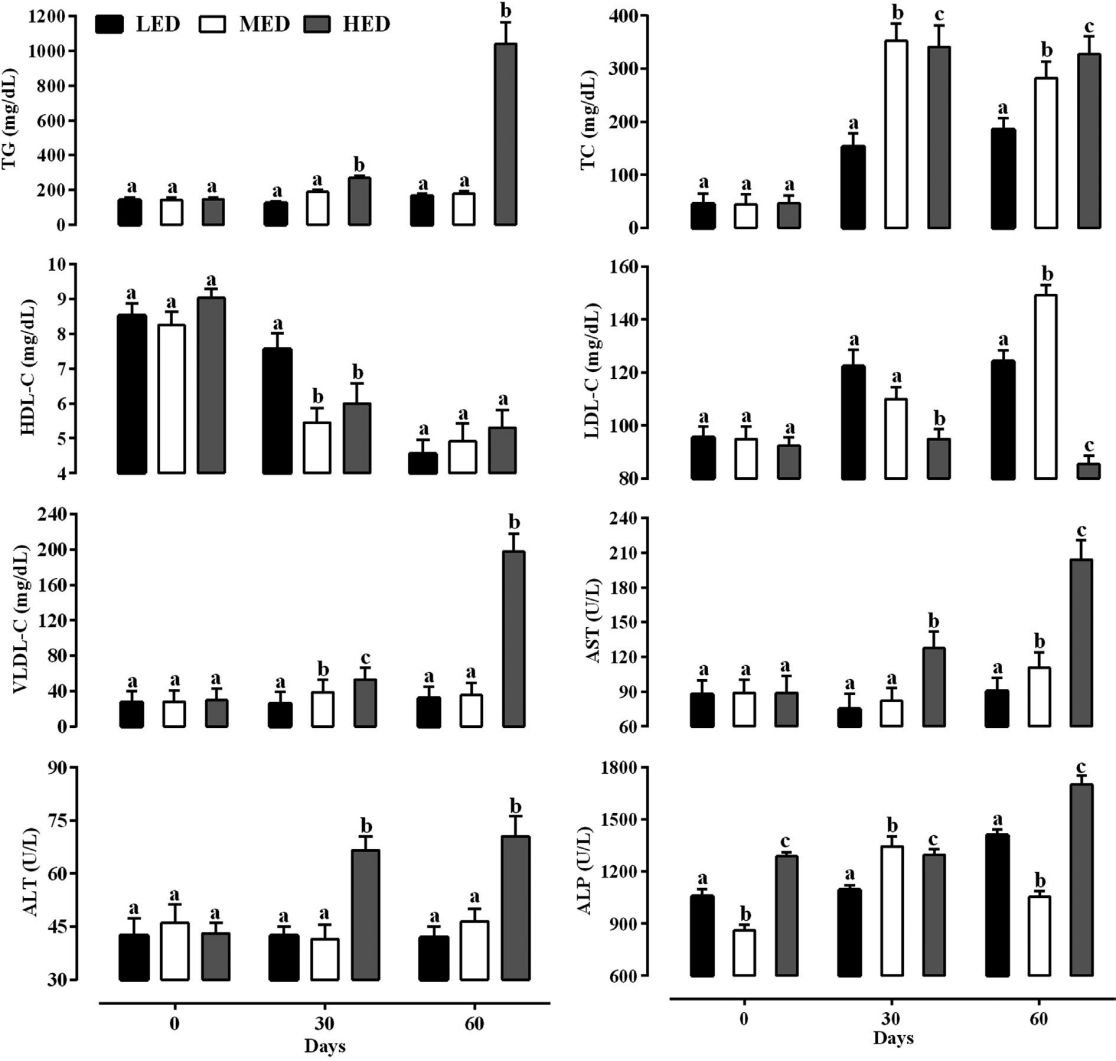
Circulating levels of lipid profile and hepatic enzymes (mean ± *SE*) in pigeons fed by three different levels of dietary energy. ALT, alanine aminotransferase; ALP, alkaline phosphatase; AST, aspartate aminotransferase; HDL, high‐density lipoprotein cholesterols; HED, high‐energy diet; LDL, low‐density lipoprotein cholesterols; LED, low‐energy diet or control group; MED, medium‐energy diet; TC, total cholesterol; TG, triglyceride; VLDL, very‐low‐density lipoprotein cholesterols. ^a,b,c^Different letters in the superscripts indicate significant differences (*p* <.05)

The serum lipid profile of pigeons fed with different levels of dietary energy is presented in Figure 1. The circulating levels of TG and VLDL in HED were significantly higher than MED and LED on days 30 and 60. HED and MED groups had higher TC compared to LED on days 30 and 60 (*p* <.05). The circulating TC in HED was significantly higher than MED on day 60. The circulating LDL decreased in HED compared to MED and LED on days 30 and 60 (*p* <.05).

In this study, the serum ALT, AST and ALP activities were measured as markers of liver health. The changes in activity of ALT, AST and ALP are shown in Figure 1. Serum activity of ALT and AST elevated in HED compared to LED and MED on days 30 and 60 (*p* <.05). There was no statistically significant difference between LED and MED in serum activity of ALT at all days of the experiment (*p* >.05). The serum activity of ALP was significantly higher in those birds assigned to the HED compared to the control diet on days 30 and 60.

**FIGURE 2:**
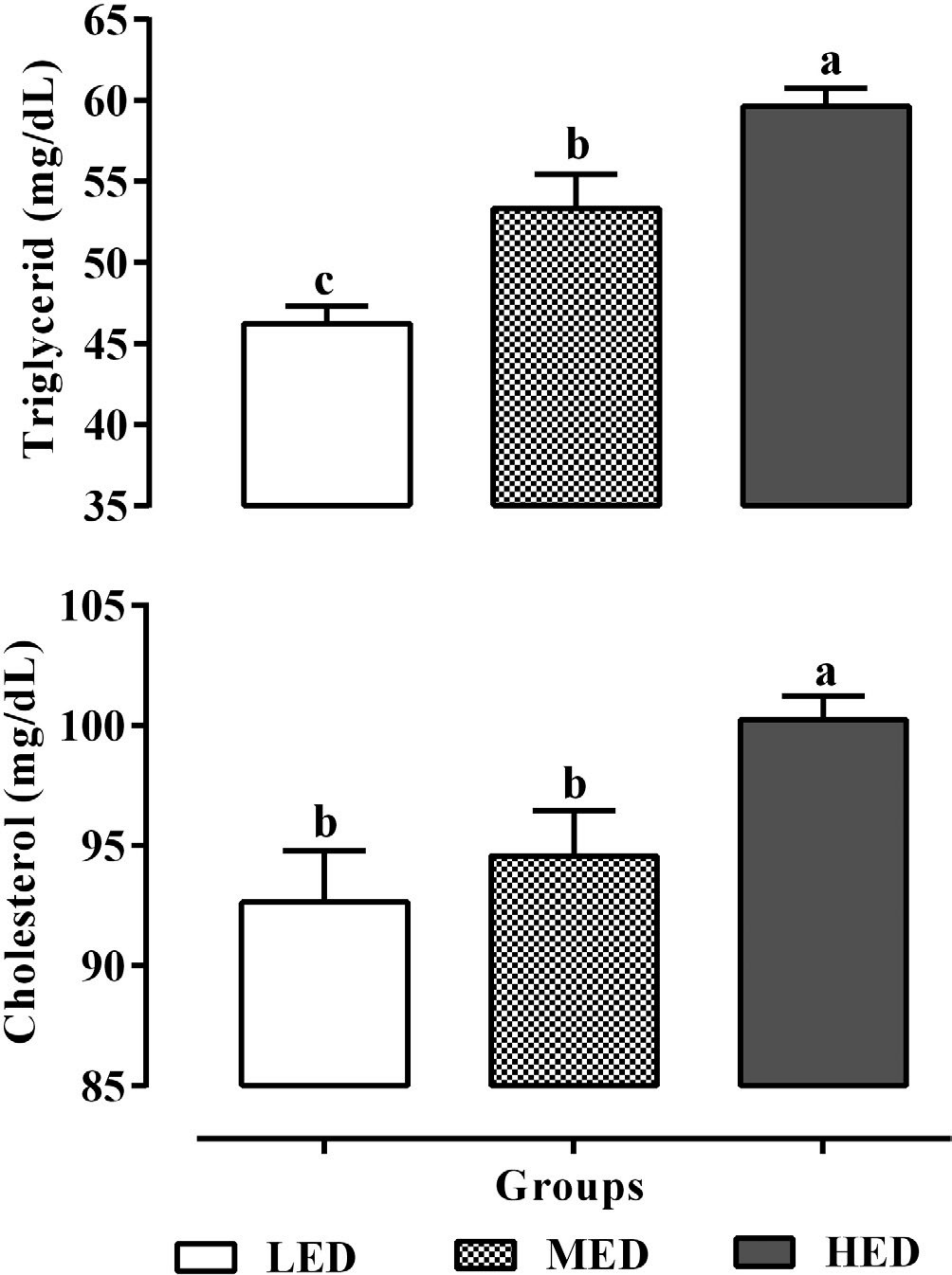
The levels of triglyceride and total cholesterol in liver tissue of pigeons fed by three different levels of dietary energy. HED, high‐energy diet; LED, low‐energy diet or control group; MED, medium‐energy diet. ^a,b,c^Different letters in the superscripts indicate significant differences (*p* <.05)

The hepatic levels of TG and TC in three different treatment groups are shown in Figure 2. Hepatic TG and TC contents in the HED group were higher than LED and MED on day 60(*p* <.05). There were no significant differences between LED and MED in hepatic TC content (*p* >.05).

Table 4 illustrates the effect of three different dietary energy levels on serum MDA, T‐AOC and cortisol in pigeons. HFD‐fed pigeon (HED) had higher MDA concentration and lower T‐AOC activity compared to LED and MED groups on day 60 (*p* <.05). The serum concentration of cortisol in the HED group was significantly higher than LED and MED. There was no significant difference between LED and MED in serum levels of MDA, T‐AOC and cortisol (*p* >.05).

**TABLE 4:** The values of MDA, T‐AOC and cortisol (mean ± *SE*) in pigeons fed by three different dietary energy at the end of study

Parameters	Group		
	LED	MED	HED
MDA (nmol/ml)	129.91 ± 8.52^a^	117.75 ± 10.15^a^	169.61 ± 10.19^b^
T‐AOC (nmol/ml)	563.43 ± 23.41^a^	538.08 ± 21.09^a^	419.31 ± 32.19^b^
Cortisol (ng/ml)	1.14 ± 0.06^a^	1.94 ± 0.26^a^	3.99 ± 0.79^b^

Note

Different letters in the superscripts (^a,b,c^) of the same row indicate significant differences (*p* <.05).

Abbreviations: HED, high‐energy diet; LED, low‐energy diet or control group; MDA, malondialdehyde; MED, medium‐energy diet; T‐AOC, total antioxidant capacity.

## 4 DISCUSSION

Excessive consumption of HFD is one of the most important causes of obesity and oxidative stress in human and laboratory animals (Chen et al., 2013; Recena Aydos et al., 2019; Yilmaz, 2012). This study revealed that the high dietary energy induced oxidative stress in domestic pigeons (Table 4). Based on obtained results, increasing dietary energy up to 3,450 kcal/kg increased serum concentration of MDA and cortisol and decreased serum levels of T‐AOC in HFD‐fed pigeons (Table 4). There were no significant differences about circulating levels of MDA, T‐AOC, and cortisol between pigeons fed by 2,850 kcal/kg and those received 3,150 kcal/kg. The obtained results from this study are in agreement with the previous study by Lasker et al., (2019) which stated that high serum levels of lipid in obese rat led to oxidative stress and ROS generation and decreased antioxidant enzyme activities. In addition, the secretion of various cytokines and hormones from adipose tissue and the phagocytic activation of nicotinamide adenine dinucleotide phosphate oxidase also play an important role in the occurrence of oxidative stress in obesity (Al‐Muzafar & Amin, 2017). It has been stated that excessive fat accumulation in adipose tissue and liver in obese animals develops a metabolic stress that disrupts physiological homeostasis and it is related to oxidative stress (Sordillo & Mavangira, 2014). Serum cortisol levels in pigeons fed by 3,450 kcal/kg were significantly higher than LED groups (*p* <.05; Table 4). Auer et al. (2016) reported that cortisol is an important index to evaluate obesity, hyperlipidaemia, fatty liver and cirrhosis in human beings. Huble et al., (2014) stated that cortisol metabolism changed in obese human beings and its regeneration from cortisone elevated within body fat mass and fatty liver. The positive relationship between hepatic fat levels and serum cortisol has been stated (Hubel et al., 2014). Based on the previous researches and this study, it may be suggested that high energy diets could induce stress and oxidative stress in pigeons which confirms the obtained results from similar studies on human and other animal models.

According to the following results, obesity was confirmed in the HED groups and it may be stated that obese pigeons suffered from stress and oxidative stress. Some parameters and indices such as BW, AWG, TWG, and FI were evaluated to investigate the effects of high‐energy‐dense diet on the development of obesity (Table 2). In this study, increasing dietary energy levels led to increase BW (17%) and TWG (73%) and decrease FI (60%) in the HED compared to LED group (*p* <.05; Table 2). Bu et al. (2015), revealed that adult pigeons fed with 2,940 kcal/kg ME had higher BW and lower FI compared to 2,630 kcal/kg. FI in avian is inversely related to the amount of dietary energy, and increasing the dietary energy value reduces FI (Abouelezz et al., 2019). Infante‐Rodríguez et al. (2016) and Zhao and Kim (2017) reported that feeding on HEDs decreased FI in broilers which is consistent with our findings. Tancharoenrat and Ravindran (2014) stated that the high level of dietary energy had no significant effects on FI in broilers. Based on the current investigation, increasing dietary energy level up to 3,450 kcal/kg led to increase weight gain and obesity in pigeons and HED groups affected with marked obesity.

As shown in Table 3, the average weight of the abdominal fat, liver and breast meat significantly increased in HED pigeons compared to the other groups. Increasing dietary energy up to 3,150 kcal/kg had no significant effects on the liver weight in MED group compared to LED. In MED pigeons, the weight of the breast muscle and abdominal fat increased by 18% and 31%, respectively, compared to LED. In HED compared to LED group, the weight of liver and abdominal fat increased significantly by approximately 21% and 47%, respectively. It may be suggested that increasing the dietary energy level up to 3,450 kcal/kg was linked to increase the weight of liver and adipose tissue in the studied birds. Various studies have been reported that the weight of the abdominal fat and breast muscle increased in birds fed fat‐rich diet due to fat deposition in these tissues (Marcu et al., 2013; Zhao & Kim, 2017). In addition, enlarged and friable liver are the most common hepatic problems in obese birds (Hochleithner et al., 2006). The weight and relative weight of abdominal fat, liver and breast muscle of pigeons fed by HEDs in this study were significantly higher than other groups which confirmed obesity in HED birds.

In this study, five common markers of dyslipidaemia, including TG, TC, HDL, LDL and VLDL were evaluated to investigate whether HED led to dyslipidaemia in pigeons (Figure 1). At the beginning of the experiment (day 0), birds had no significant differences in serum levels of lipid profiles. Circulating TG increased in HED compared to LED and MED groups at all days, and the serum TG levels in MED group were higher than LED at days 15 and 45 (*p* <.05). As Figure 1 shows, the serum concentrations of TG significantly increased (approximately six‐fold) in the HED compared to LED and MED groups at day 60. In the HED group, TG levels at day 60 (1,040.50 ± 5.72 mg/dl) significantly increased approximately seven times compared to day 0 (147.00 ± 1.51 mg/dl). Serum levels of TC significantly increased in MED and HED groups compared to LED group during the experiment. The HED group had the most values of TC among the various groups at day 60. Circulating levels of TC significantly increased more than six times at day 60 compared to day zero in MED and HED groups. The results indicated that high‐fat intake in pigeons was associated with disorder in lipid metabolism such as increased serum TG, TC and LDL, which are the most important indices of obesity in birds (Godea (Lupei) et al., 2020). Many experimental studies have been reported that dyslipidaemia is present in broilers following high‐energy density diet consumption (Wenqing et al., 2017; Zhao & Kim, 2017). In the present experiment, no significant and consistent change was found in the circulating HDL among the different groups. In contrast, some previous studies indicated that the serum levels of HDL increased in birds fed with HFD (Ge et al., 2019; Zhang et al., 2008). HED group had the lower LDL and higher VLDL compared to other groups at different times. Ge et al. (2019) reported that high dietary energy decreased the serum LDL concentrations in broilers. Zhao and Kim (2017) confirmed that the serum levels of LDL in broilers increased following feeding by a fat‐rich diet. Increasing dietary energy levels up to 3,450 kcal/kg in pigeons led to dyslipidaemia and a significant increase in the serum levels of TG (84%), TC (43%) and VLDL (83%) compared to control diet.

High‐energy diets may induce hepatic fat accumulation, fatty liver and hepatic disorders. Previous studies stated that HED elevated the serum levels of TG and TC, which developed lipotoxicity, fat accumulation in the liver, and hepatic damage in human (Alkhouri et al., 2009). There are several circulating hepatic biomarkers to evaluate hepatic healthiness and performance. Generally, the AST, ALT and ALP enzymes are common hepatic indices and are release from damaged hepatocytes and transport to the bloodstream. So, evaluating the serum activity of these enzymes is valuable to assess hepatic health in birds (Senanayake et al., 2015; Zhang et al., 2008). In this study, serum activities of AST and ALT were significantly higher in pigeons fed by 3,450 kcal/kg compared to birds on lower energy on day 60 (Figure 1). In the HED group, AST and ALT increased significantly by approximately 2 and 1.5‐fold at day 60 compared to day 0, respectively. Zhang et al., (2008) confirmed that the serum levels of AST and ALT were increased by approximately two‐fold in broilers following feeding HFD. In this study, serum activity of ALP elevated significantly in HED group. Various researchers showed that serum activities of ALT, AST and ALP elevated in fatty liver syndrome in chicken (Rozenboim et al., 2016; Zhang et al., 2008). As expected, in this study, obese pigeons had high levels of AST, ALT and ALP than the other birds and it may be suggested that obesity may lead to hepatic damage and dysfunction.

Lipid accumulation in the hepatocytes is the most remarkable feature of fatty liver in numerous animal models (Song et al., 2017). In this study, the TG and TC contents of the liver tissue increased significantly in pigeons fed with 3,450 kcal/kg ME compared to birds on lower dietary energies (Figure 2) that were consistent with some previous studies (Rozenboim et al., 2016; Song et al., 2017). Bu et al., (2015) showed that increasing dietary energy levels up to 2,940 kcal/kg ME led to an increase the weight of the pigeons' liver, but it did not lead to fat accumulation in the liver but in this study, 3,450 kcal/kg ME led to an increase fat accumulation in hepatocytes in pigeons.

## 5 CONCLUSION

In conclusion, this study showed that increasing dietary energy up to 3,450 kcal/kg led to obesity and oxidative stress in pigeons. Accordingly, it could be stated that HEDs and obesity induce oxidative stress in pigeons and controlling the dietary energy intake of pigeons is necessary to prevent oxidative stress in them.

## CONFLICTS OF INTEREST

The authors declare no conflict of interest.

## AUTHOR CONTRIBUTION

**Seyyedeh Alemeh Hosseinian:** Conceptualization; Data curation; Formal analysis; Funding acquisition; Investigation; Methodology; Project administration; Resources; Software; Supervision; Validation; Visualization; Writing‐original draft; Writing‐review & editing. **Fereshteh Hasanzadeh:** Data curation; Investigation; Software; Writing‐original draft.

## ANIMAL ETHICAL STATEMENT

All protocols used in the study were approved by the Iranian Animal Ethics Standards under the supervision of the Iranian Society for the prevention of cruelty to animals and Shiraz University Research Council (IACUC no: 4687/63).

### PEER REVIEW

The peer review history for this article is available at https://publons.com/publon/10.1002/vms3.478.
